# Tunable absorber embedded with GST mediums and trilayer graphene strip microheaters

**DOI:** 10.1038/s41598-021-83304-y

**Published:** 2021-02-11

**Authors:** M. Pourmand, P. K. Choudhury, Mohd Ambri Mohamed

**Affiliations:** Institute of Microengineering and Nanoelectronics, Universiti Kebangsaan Malaysia, UKM, 43600 Bangi, Selangor Malaysia

**Keywords:** Optics and photonics, Applied optics, Optical materials and structures, Other photonics

## Abstract

Investigation was made of the optical response of metal-dielectric stacks-based cavity structures embedded with graphene microheaters for the purpose of perfect absorption. The absorber configuration exploits the Ge_2_Sb_2_Te_5_ (GST) phase changing medium, and the effects of different parametric and operational conditions on the absorption spectra were explored. The refractive indices of GST layers can be manipulated by the external electrical pulses applied to microheaters. The amplitude and duration of electrical pulses define the crystallinity ratio of the used GST mediums. The results revealed achieving perfect absorption (> 99%) in the visible and infrared (IR) regimes of the electromagnetic spectrum upon incorporating two thin GST layers of different thicknesses (in the stack) in the amorphous state. The proposed configuration showed the capability of introducing independent transition state (amorphous and/or crystalline) for each GST layer—the visible regime could be extended to the IR regime, and the perfect absorption peak in the IR regime could be broadened and red-shifted. It is expected that the structure would find potential applications in active photonic devices, infrared imaging, detectors and tunable absorbers.

## Introduction

Investigation of the electromagnetic behavior of metal-dielectric stacks-based cavity systems attracted significant attention by the R&D community toward realizing perfect absorption in the range extended from the visible light to the infrared (IR) regime^1–4^. This is because of their ability to provide scalable and cost-effective schemes to devise photonic components for varieties of technological applications^5^. The significance of metamaterials and plasmonic absorbers remains primarily due to the possibility of achieving near-to-perfect absorption (> 99%) with superb control over the spectral characteristics^6–13^, thereby making them potential candidates for building blocks of future optical systems with enormous applications, such as energy harvesters^14–16^, IR imaging systems^17^, label-free sensors^18^, etc. Attempts have been made to broaden the absorption spectrum by employing different strategies^8–11,16^ and techniques, such as exploiting the hyperbolic metamaterials^8,18^ or multiplexed patterned metallic structures^19^. However, the complexity of nanofabrication processes, scarcity of natural materials^19–22^ and incompatibility with large-scale manufacturing remain the determining factors for relatively less use of these in the terahertz (THz) and IR regimes.

Tunable materials have been attractive in designing active metamaterials and plasmonic absorbers. The integration of tunable materials, such as graphene^17,23–25^ and phase changing mediums (PCMs)^26–30^, into the design of active metamaterials allows them to be useful in many photonic applications. Within the context, graphene- and liquid crystal-based devices are tuned by external electrical stimulus, whereas the PCMs (such as the VO_2_ and G_2_Se_2_Te_5_ (GST) mediums)-based devices can be tuned electrically^26^, thermally^29^, and/or optically^31,32^. These mediums have been mostly used in designing absorbers^20,22,29,30^. Ghosh et al.^33,34^ reported almost unity absorption over a broad bandwidth in the THz and low IR regimes where graphene shows metallic behavior. They employed patterned graphene layers on the top of the dielectric-metal stack to enhance the absorption characteristics. However, this approach is not suitable in the UV and visible regimes.

Among the PCMs, the VO_2_-based systems are more useful to exhibit perfect absorption in the IR wavelengths, whereas the GST-based systems provide nearly perfect absorption in the IR and visible light regimes. However, the major drawback of VO_2_ medium is the phase transition that happens at a relatively low temperature (68 °C) under continuous temperature control^35,36^. On the other hand, the phase of GST remains stable in maintaining the primary state—the amorphous or crystalline—even in the absence of control stimulus. More importantly, GST is extremely scalable, and can be easily integrated in technology-based devices of commercial potentials.

Microheating systems have been of significant importance in various need-based miniaturized photonic devices owing to exhibiting high Joule heating performance—the ability of providing adequate heat in minuscule Joules^37,38^. Taghinejad et al*.*^38^ reported that ITO-based microheaters can be employed to realize fast and reversible switching between the two GST states by applying a low voltage (~ 20 V). The authors demonstrated that applying 200 ms electrical pulses of different amplitudes gradually decreases the resistivity of GST layer from high (the amorphous state) to low (the crystalline state) values^38^. They determined that the intermediate crystalline states can be configured through adjusting the excitation pulse amplitude.

The quality of microheating systems relies on the key factor to achieve high Joule heating performance that essentially depends on the low resistivity value of heater. The ITO has a sheet resistance of approximately 100 Ω/sq—the value which can heat the monolayer graphene having a sheet resistance of 10 kΩ/sq. However, the ITO requires rapid thermal annealing process in which the thin film undergoes heating to a high temperature of 450 °C for ~ 15 min^38^. This process also reduces the optical loss of ITO layer to a value < 20%, which is very high, as compared to graphene that shows the optical loss of about 2.7% only. In addition, the ITO suffers from many other issues, such as being expensive, having difficulties in patterning, easily being wore out or cracked^39^. The ITO can be replaced with graphene owing to its being a transparent and flexible material, which can be easily patterned by exploiting lithography techniques. Apart from this, the low intrinsic optical loss, high in-plane thermal conductivity, and low heat capacity remain the additional advantages^39^.

Investigators have reported various techniques to exploit graphene as the microheater for GST mediums^40–43^. In this stream, Rios et al*.*^37^ demonstrated the optoelectronic framework incorporating undoped monolayer graphene heater to perform reversible and controllable switching between four different levels of crystallinity for a large area of GST medium. Ref.^41^ discusses the techniques to enhance the Joule performance of graphene heater, and reports on decreasing the sheet resistance and minimizing the contact resistance between graphene layer and electrodes. Within the context, the sheet resistance of graphene is inversely related to its bidimensional conductivity—the factor that can be defined by the electron relaxation time and the numbers of layers^33,42^. Hung et al*.*^42^ showed that increasing the number of graphene layers up to 4, thereby contributing to nearly 1 nm total thickness, would increase the Joule performance; a high temperature up to 600 °C can be achieved at a low voltage of 4.3 V. However, they showed that the use of graphene layer thickness > 1 nm slightly increases the sheet resistance (of heater) since the increased edge structure would reduce the heating efficiency. Luo et al*.*^43^ demonstrated that a high lattice temperature up to 1397 °C can be achieved by encapsulating the graphene layers with hexagonal boron nitride.

In view of the use of graphene layer(s) as microheater for GST medium, the present work is aimed at investigating the tunable absorption characteristics of a specially designed multilayered planar structure that incorporates graphene as well as GST. In particular, we use metal-dielectric-based cavity structures embedded with two graphene microheaters each having a thickness of 1 nm, which is corresponding to nearly a trilayer graphene sheet. The refractive indices of GST layers can be manipulated by the external electrical pulses applied to graphene microheater layers. We obtain the simulated/numerical results by using the equivalent circuit model in Ref.^42^. We also present the optimum dimensions of the geometrical parameters that would yield high absorption (by the structure) in the visible and IR regimes of the electromagnetic spectrum. The study includes the effect of operational conditions on the absorption characteristics by exploiting the transfer matrix method (TMM). The effect of two different phases of GST (amorphous and crystalline) on the absorption characteristics are also studied. The obtained results demonstrate achieving dual broad-band absorption spectra—the feature that would be promising in many optical applications, such as bolometer, color displays, IR detectors, tunable camouflages, energy harvesters and optical communications.

## Design and analysis

**Figure 1:**
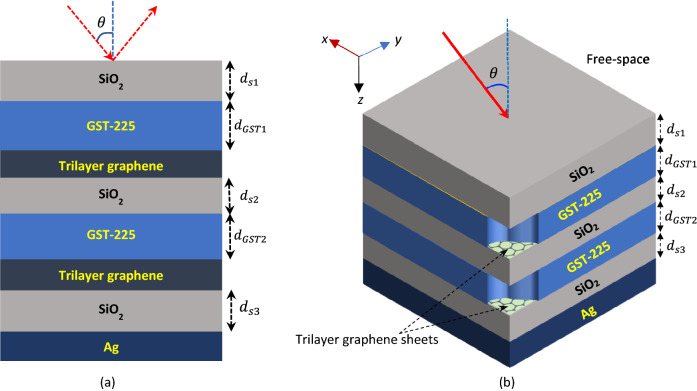
(**a**) 2D and (**b**) 3D schematics of the proposed GST-graphene-SiO_2_ mediums-based multilayered planar structure.

Figure 1 exhibits the schematic of the proposed multilayered planar structure comprised of 08 layers altogether; Fig. 1a, b, respectively, show the 2D and 3D versions of the configuration. Here the bottom is a silver (Ag) layer having a thickness of 50 nm. Also, it has 02 top-up stacks of GST-graphene-SiO_2_ mediums as space layers, and finally, a thin layer of SiO_2_ on top to serve the purpose of capping. This protects the upper planar GST medium from evaporation as well as provides more heat confinement within the structure. We use the SiO_2_ medium because of its stable interaction with graphene sheets, and also, low amount of loss in the IR regime^44^. The bottom silver layer acts as a reflective mirror which introduces critical coupling conditions.

The proposed multilayered structure can be fabricated exploiting the chemical vapor deposition (CVD) technique without undergoing complex lithography process. Multilayered graphene medium can be deposited over a thin layer of SiO_2_^23,25,37^. A graphene medium having 1 nm thickness normally corresponds to trilayer graphene structure, which can be synthesized on a ceramic substrate by the CVD method, and then transferred on the SiO_2_ layer^42^. We consider the trilayer graphene sheets as microheaters for the used GST layers. The bottom Ag layer can be deposited over the Si substrate by thermal evaporation. The same process can be exploited to deposit the GST layers on the SiO_2_ mediums^38,41,42^. It has been reported before that 100 nm thick Ti/Au metal pads on graphene layers can be used as metal connectors to induce the required electrical current for heating the GST layers^37,41^.

**Figure 2:**
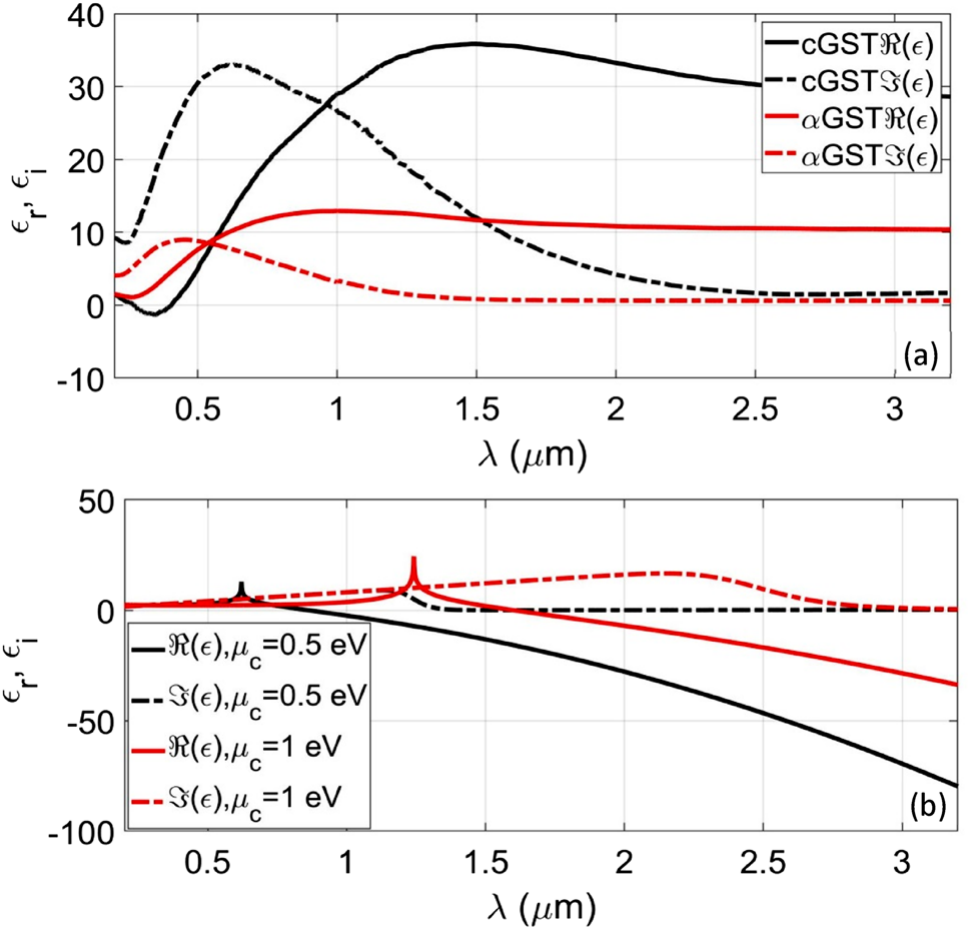
Wavelength dependence of permittivity for (**a**) Ge_2_Se_2_Te_5_ in the ${\upalpha }$*-* and *c*-phases, and (**b**) monolayer graphene corresponding to the chemical potential ${\upmu }_{{\text{c}}} = 0.5$ eV and ${\upmu }_{{\text{c}}} = 1.0$ eV.

Using the optical constants of mediums^23,26,45^, Fig. 2a exhibits the variations of the real ($\epsilon_{r}$) and imaginary ($\epsilon_{i}$) parts of the permittivity of GST in the amorphous ($\alpha$) and crystalline (*c*) states with wavelength. These components of permittivity are, respectively, shown by $\Re (\epsilon)$ and ${\Im (\epsilon)}$. It is clear from this figure that the permittivity of both the phases of GST greatly depends on the operating wavelength. However, the dependence on wavelength is more prominent up to 2.5 µm in the case of $c$-phase, whereas the $\alpha$-phase exhibits it up to 1.5 µm only. Beyond these operating points, the permittivity values of the *c*- and $\alpha$-phases of GST become almost independent of wavelength. Though in both the phases the real part of permittivity is always larger than the imaginary part, the values of those become very close in the $c$-phase above 2.5 µm wavelength.

On the other hand, the conductivity $\sigma \left( \omega \right)$ of N-layer graphene can be determined as^46,47^

1 $$\sigma \left( \omega \right) = \frac{{2e^{2} k_{B} T}}{{\pi \hbar^{2} N}}\left\{ {\frac{1}{\gamma - j\omega }} \right\}\mathop \sum \limits_{m = 1}^{N} \ln \left[ {2\cosh \left( {\frac{{\mu_{c} + 2\alpha_{1} \cos m\pi /N + 1}}{{2k_{B} T}}} \right)} \right]$$

where $\hbar$ ($= h/2\pi$) is the reduced Planck’s constant, $k_{B}$ is Boltzmann’s constant, $T$ is the absolute temperature, $e$ is the electronic charge, $\gamma$ is the rate of scattering (which is inversely proportioned to the relaxation time $\tau$), $N$ is the number of graphene layers and $\alpha_{1}$ (= 217 meV) is the interaction energy of misoriented graphene layers^47^. In the present work, we take $\gamma = 1.32$ meV. It has been reported before that, in the case of N-layer graphene, a higher carrier density can be obtained, thereby demanding a lower external electrical potential^48^.

Figure 2b exhibits the wavelength dependence of $\epsilon_{r}$ and $\epsilon_{i}$ in the case of monolayer graphene (i.e., $N = 1$). We observe in this figure that the real part shows the positive and negative dependence on the operating wavelength, whereas the imaginary part remains positive-valued only. The increase in chemical potential $\mu_{c}$ causes red-shifts in the peaks of the real and imaginary parts, thereby determining $\mu_{c}$ to be the tuning parameter to alter the optical characteristics of graphene layer.

We study the propagation of electromagnetic waves through the proposed structure using a TMM-based numerical approach^30,48^. In the treatment, we consider plane waves of either transverse electric (TE) polarization with ${\varvec{E}} = \left( {0,E_{y} ,0} \right)$ and ${\varvec{H}} = \left( {H_{x} ,0,H_{z} } \right)$ or transverse magnetic (TM) polarization with ${\varvec{E}} = \left( {E_{x} ,0,E_{z} } \right)$ and ${\varvec{H}} = \left( {0,H_{y} ,0} \right)$ impinge on the top surface of the proposed structure at an angle $\theta$ (Fig. 1). We consider the multilayer stack as a uniaxial homogeneous medium, which has the optical axis perpendicular to the plane of interface, i.e., the *x*–*y* plane. Now, the wavenumbers corresponding to the TE- and TM-polarized waves propagating in the $i$ th layer of the structure can be determined as^49^

2 $$k_{iz} = \pm \sqrt {\epsilon_{i,t} k_{0}^{2} - k_{ix}^{2} } \quad ({\text{for the TE-polarized incidence}})$$

3 $$k_{iz} = \pm \sqrt {\epsilon_{i,t} k_{0}^{2} - \frac{{\epsilon_{i,t} }}{{\epsilon_{i, \bot } }}k_{ix}^{2} } \quad ({\text{for the TM-polarized incidence}})$$

In these equations, $k_{0}$ is the free-space wavenumber,$k_{iz}$ is the wavenumber of the normal components, $k_{ix} = k_{0} \sin \theta$, and the subscripts $t$, $\bot$ represent the transverse and normal (directions) permittivity values, respectively.

We now exploit Maxwell’s equations to satisfy the boundary conditions at the two interfaces of each layer. Then the equations are reorganized in the form of a $2 \times 2$ matrix, i.e., the transfer matrix $M_{i}$ describing the propagation characteristics corresponding to the TE- and TM-polarizations, as follows^48^:

4 $$M_{i} = \left[ {\begin{array}{*{20}c} {\cos \left( {k_{iz} d_{i} } \right)} & { - \frac{j}{{p_{i} }}\sin \left( {k_{iz} d_{i} } \right)} \\ { - {\text{j}}p_{i} \sin \left( {k_{iz} d_{i} } \right)} & {\cos \left( {k_{iz} d_{i} } \right)} \\ \end{array} } \right]$$

In Eq. (4), $d_{i}$ is the thickness of each layer, $\mu_{0}$, $\epsilon_{0}$, are the respective free-space values of permittivity and permeability, $\epsilon_{i}$ is the permittivity of the $i$ th layer, and $p_{i}$ is determined by the wave polarizations so that it assumes the values equal to either $\left( {k_{iz} /\omega \mu_{0} } \right)$ or $\left( {k_{iz} /\omega \mu_{0}\epsilon_{0}\epsilon_{i} } \right)$, respectively, corresponding to the cases of TE- or TM-polarizations.

For an N-layer model, the total transfer matrix ($M_{T}$) of the structure (of Fig. 1) is evaluated as a serial product of transfer matrices $M_{i}$ ($i = 1,2,3, \ldots ,N$), i.e.,

5 $$M_{T} = \mathop \prod \limits_{i = 1}^{N} M_{i} = \left[ {\begin{array}{*{20}c} {m_{11} } & {m_{12} } \\ {m_{21} } & {m_{22} } \\ \end{array} } \right]$$

where $N$ is the total number of component layers and $m_{ij}$ represents the transfer matrix components.

The reflection $R$ and transmission $T$ coefficients corresponding to the cases of TE- and TM-polarized incidence excitations can be obtained as^49^:

6 $$R = \left| r \right|^{2} = \left| {\frac{{\left( {m_{11} + m_{12} p_{N + 1} } \right)p_{0} - m_{21} - m_{22} p_{N + 1} }}{{\left( {m_{11} + m_{12} p_{N + 1} } \right)p_{0} + m_{21} + m_{22} p_{N + 1} }}} \right|^{2}$$

7 $$T = \left\{ {\begin{array}{*{20}l} {\left| {\frac{{2p_{0} }}{{\left( {m_{11} + m_{12} p_{N + 1} } \right)p_{0} + m_{21} + m_{22} p_{N + 1} }}} \right|^{2} } \hfill & {({\text{for the TE case}})} \hfill \\ {\frac{{p_{0} }}{{p_{N + 1} }}\left| {\frac{{2p_{0} }}{{\left( {m_{11} + m_{12} p_{N + 1} } \right)p_{0} + m_{21} + m_{22} p_{N + 1} }}} \right|^{2} } \hfill & {({\text{for the TM case}})} \hfill \\ \end{array} } \right.$$

In Eqs. (6) and (7), $p_{0}$ and $p_{N + 1}$ are evaluated in accordance with the incidence wave polarization. In the case of TE-polarization, we have $p_{0} = \sqrt {\varepsilon_{0} /\mu_{0} } \cos \theta_{0}$ and $p_{N + 1} = \sqrt {\varepsilon_{N + 1} /\mu_{N + 1} } \cos \theta_{N + 1}$, whereas corresponding to the TM-polarized incidence, we have $p_{0} = (\sqrt {\varepsilon_{0} /\mu_{0} } ){/}\cos \theta_{0}$ and $p_{N + 1} = (\sqrt {\varepsilon_{N + 1} /\mu_{N + 1} } ){/}\cos \theta_{N + 1}$. In these equations, the subscripts $N + 1$ and 0 represent the situations at the input and output layers, respectively.

The absorbance (or the absorption coefficient) $A$ for both the polarizations can be obtained in the form $A\left( \lambda \right) = 1 - \left[ {T\left( \lambda \right) + R\left( \lambda \right)} \right]$; $T\left( \lambda \right)$ and $R\left( \lambda \right)$ being the wavelength-dependent transmission and reflection coefficients, respectively. In the case of critical coupling, the transmission coefficient is eliminated, thereby the absorbance to assume the form as $A\left( \lambda \right) = 1 - R\left( \lambda \right)$. The critical coupling occurs when the rate of reflection becomes equal to the rate of absorption. In this view, the perfect absorption can be attained at the resonance frequencies. Many investigations have been performed to enhance the perfect absorption in the optical regime by using the Fabry–Perot cavity structures and lossy dielectric mediums^1,3,29,42^. Within the context, one may think of the effects due to the cross-polarized component, which may alter the total absorptivity^50^. However, we neglect the cross-polarization effect because the medium is considered as uniaxial homogeneous, as stated before.

## Results and discussion

**Figure 3:**
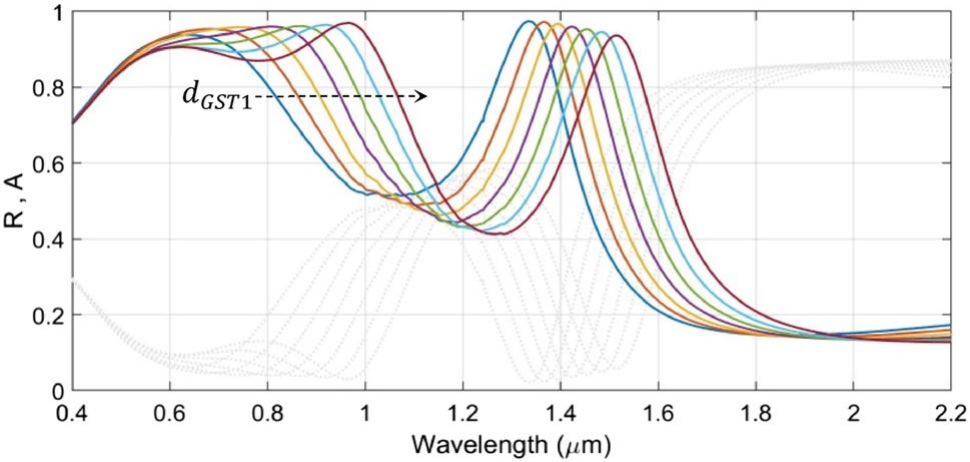
Wavelength dependence of the absorbance ${\text{A}}$ (and reflectance ${\text{R}}$) of the structure under fixed ${\text{d}}_{{{\text{GST}}2}}$ (= 90 nm), ${\text{d}}_{{{\text{s}}1}}$ (= 100 nm), and ${\text{d}}_{{{\text{s}}2}} = {\text{d}}_{{{\text{s}}3}}$ (= 50 nm), and varying ${\text{d}}_{{{\text{GST}}1}}$. Coloured lines correspond to absorbance; gray dotted lines represent reflectance. The dashed arrow represents increase in ${\text{d}}_{{{\text{GST}}1}}$.

We now investigate the tunable absorption characteristics of the proposed structure under various operating conditions. In this stream, we first take up the influence of the upper GST layer thickness $d_{GST1}$ in the amorphous phase, and vary this in a range of 90–150 nm at a step of 10 nm, keeping the value of the thickness $d_{GST2}$ of the lower GST layer fixed (to a value of 90 nm). Also, we take the other parametric values as $d_{s1} = 100$ nm and $d_{s2} = d_{s3} = 50$ nm (Fig. 1). We use trilayer graphene at this stage having a thickness of 1 nm and a chemical potential of $\mu_{c} = 0.1$ eV. Figure 3 exhibits the variation of absorbance $A$ (and also, the reflectance $R$) with wavelength considering the TM-polarized incidence excitation impinging on the top SiO_2_ surface normally (i.e., $\theta = 0^{ \circ }$).

Figure 3 shows that the absorption (and reflection) characteristics of the proposed structure have two spans of resonances, viz. the visible (600–1000 nm) and IR (1300–1570 nm) regimes. This figure exhibits that the increase in the thickness of the upper GST layer (i.e., $d_{GST1}$), as shown by the dashed arrow in the figure (the direction of arrow represents increase in $d_{GST1}$), results in significant broadening of the resonance absorption in the visible regime with a *perfect* absorption (> 99%). Moreover, we observe red-shifts in the absorption peak upon increasing the value of $d_{GST1}$—the feature that remains more prominent in the visible regime than the IR span.

**Figure 4:**
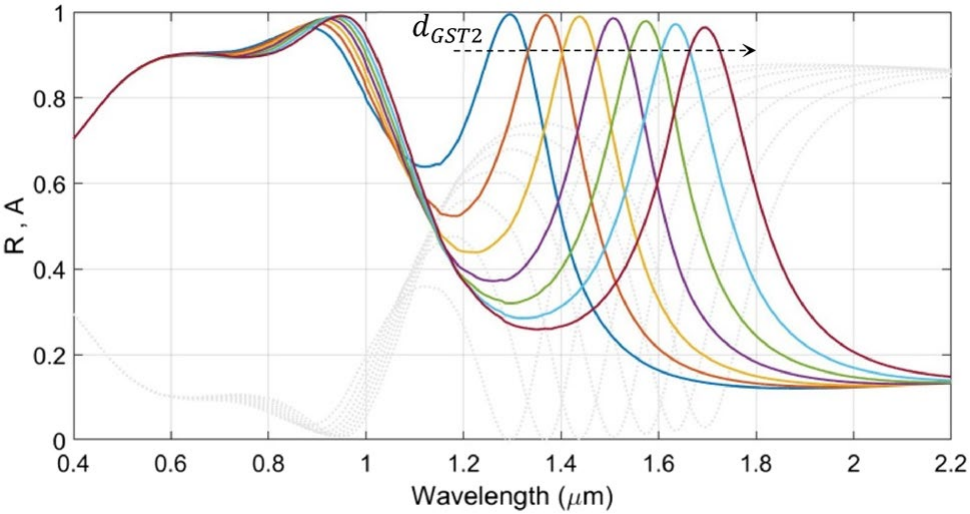
Wavelength dependence of the absorbance ${\text{A}}$ (and reflectance ${\text{R}}$) under fixed ${\text{d}}_{{{\text{GST}}1}}$ (= 130 nm) and varying ${\text{d}}_{{{\text{GST}}2}}$; the other geometrical parameters are as used in Fig. 1. Coloured lines correspond to absorbance; gray dotted lines represent reflectance. The dashed arrow represents increase in ${\text{d}}_{{{\text{GST}}2}}$.

We now consider the effect of varying the value of $d_{GST2}$ in a range of 50–110 nm, keeping $d_{GST1}$ fixed (130 nm), on the absorbance and reflectance. The other parametric/operational conditions are left unchanged as before. We suppose that, due to shorter optical length of the lower cavity, the thickness of the lower GST layer (i.e., $d_{GST2}$) would be shorter than that of the upper cavity. Figure 4 depicts the results of this study.

We clearly observe in Fig. 4 that the alterations in the thickness of the lower GST layer (i.e., $d_{GST2}$) has significant impact on the absorption peaks in the IR regime, whereas those in the visible range remain almost intact, so far as the broadening of absorption spectra is concerned. We observe that, in the IR regime, the increase in $d_{GST2}$ (as shown by the dashed arrow in the figure) causes the peak absorption to decrease a little, and also, the absorption peaks undergo strong red-shifts from 1295 to 1696 nm. Following Fig. 4, in order to maintain strong absorption, we keep $d_{GST2} = 70$ nm, in order to achieve the maximum absorption at the desirable wavelength of about 1.45 μm. Within the context, it is worth mentioning that the red-shifts in absorption peak are linearly scaled for a larger GST layer thickness. Since most of the environmental gas sensors operate in the mid-IR regime, this property remains desirable for designing tunable optical gas sensors^51^.

**Figure 5:**
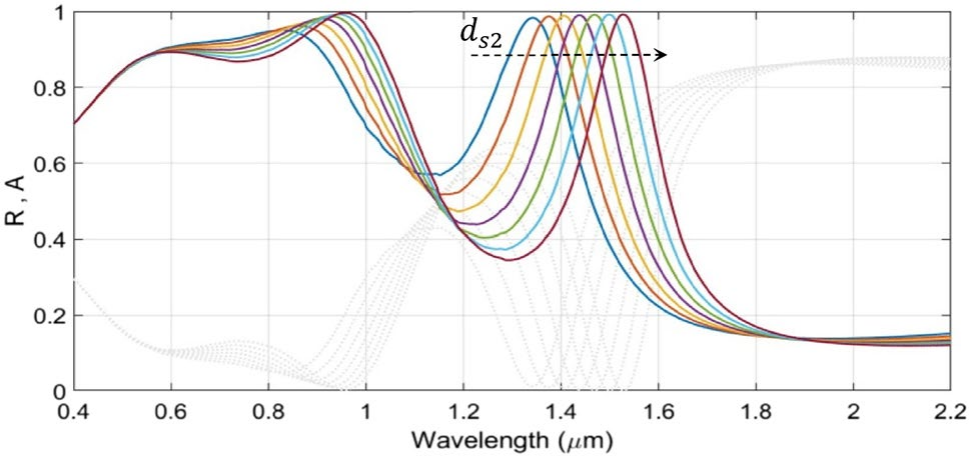
Plots of absorbance ${\text{A}}$ (and reflectance ${\text{R}}$) against wavelength under fixed values of ${\text{d}}_{{{\text{GST}}1}}$ (= 130 nm) and ${\text{d}}_{{{\text{GST}}2}}$ (= 70 nm), and varying values of ${\text{d}}_{{{\text{s}}2}}$; the other parameters are as used Fig. 1. Coloured lines correspond to absorbance; gray dotted lines represent reflectance. The dashed arrow represents increase in ${\text{d}}_{{{\text{s}}2}}$.

Next, we attempt to study the effect of SiO_2_ layer thicknesses. To perform this, we first sweep the value of the upper SiO_2_ layer thickness $d_{s2}$ in the range of 50–150 nm, keeping the upper and lower GST layer thicknesses as $d_{GST1} = 130$ nm and $d_{GST2} = 70$ nm, respectively; the other parametric and operational conditions are used as before. Figure 5 exhibits the results of this investigation, which essentially reveals the linear relationship between $d_{s2}$ and the obtained red-shifts in the absorption peaks (with increasing $d_{s2}$, as shown by the dashed arrow in Fig. 5). Interestingly, the absorption spectra in the visible and IR regimes exhibit very small increase in the absorption peaks, and reach *perfect* absorption condition corresponding to the largest chosen value of $d_{s2}$ (i.e., 150 nm). We also observe that, in the visible regime, the increase in $d_{s2}$ results in wider absorption band, which is in contrast to what we observe in the IR range, wherein the width of the absorption band gradually decreases with increasing $d_{s2}$.

**Figure 6:**
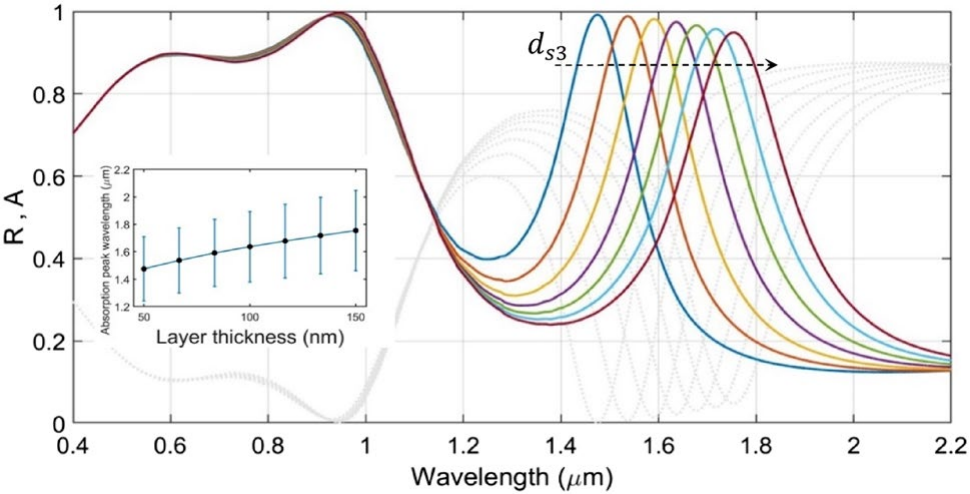
Plots of absorbance ${\text{A}}$ (and reflectance ${\text{R}}$) against wavelength under fixed values of ${\text{d}}_{{{\text{GST}}1}}$ (= 130 nm), ${\text{d}}_{{{\text{GST}}2}}$ (= 70 nm), and ${\text{d}}_{{{\text{s}}2}}$ (= 120 nm), and varying values of ${\text{d}}_{{{\text{s}}3}}$; the other parameters are used as before. Coloured lines correspond to absorbance; gray dotted lines represent reflectance. The dashed arrow represents increase in ${\text{d}}_{{{\text{s}}3}}$. The inset diagram compares the different absorption spans using different values of ${\text{d}}_{{{\text{s}}3}}$.

We now focus on the effect of the lower SiO_2_ layer thickness $d_{s3}$ on the absorption spectra. To investigate this, we vary $d_{s3}$ in a range of 50–150 nm, while keeping the other parametric/operating conditions fixed, as considered before. We take the values of $d_{GST1}$, $d_{GST2}$ and $d_{s2}$ as 130 nm, 70 nm and 120 nm, respectively. Figure 6 illustrates the results of this investigation, wherein we notice that the increase in $d_{s3}$ causes significant impact on the absorption characteristics in the IR regime; the effect in the visible regime remains the least. This is attributed to the fact that the absorption in cavities is mostly caused by the GST layers. As the thickness of the upper SiO_2_ layer increases, the optical length of cavity also undergoes increase, thereby resulting in red-shifts of the absorption peaks. However, the thicker the SiO_2_ layer is, the thinner the GST layer becomes in a cavity, which results in lower absorption property. We also observe that the absorption span gradually increases with increasing thicknesses of the SiO_2_ layer—the feature that the inset of Fig. 6 depicts. Based on the obtained results, we choose $d_{s3} = 60$ nm in further investigations as the respective absorption peak exists close to 1.5 μm wavelength.

**Figure 7:**
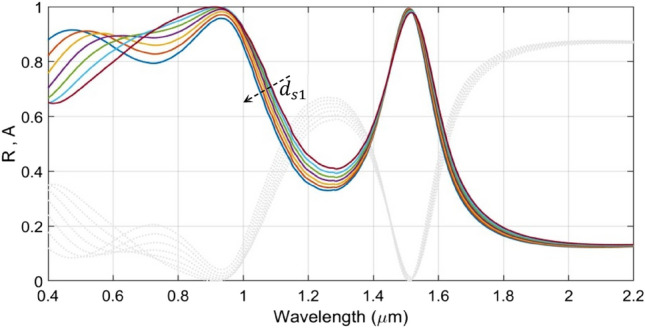
Plots of absorbance ${\text{A}}$ (and reflectance ${\text{R}}$) versus wavelength under fixed values of ${\text{d}}_{{{\text{GST}}1}}$ (= 130 nm), ${\text{d}}_{{{\text{GST}}2}}$ (= 70 nm), ${\text{d}}_{{{\text{s}}2}}$ (= 120 nm), ${\text{d}}_{{{\text{s}}3}}$ (= 60 nm) and varying ${\text{d}}_{{{\text{s}}1}}$. Coloured lines correspond to absorbance; gray dotted lines represent reflectance. The dashed arrow represents increase in ${\text{d}}_{{{\text{s}}1}}$.

We now examine the effect of the capping layer thickness $d_{s1}$ on the absorption characteristics. This thickness can be kept in a range of 70–130 nm. However, the afore discussed results use $d_{s1} = 100$ nm as a default value, as stated before. Figure 7 shows the wavelength-dependence of absorption (and reflection) by the structure under varying $d_{s1}$. We observe in this figure that the increase in capping layer thickness causes enhancement in absorption in the visible regime. The figure exhibits *perfect* absorption, while the span is decreased upon increasing $d_{s1}$. In the IR regime, however, the spectral characteristics are not appreciably affected, thereby almost eliminating red-shifts in the absorption peaks—the feature in contrast to what noticed before. As such, in the 70–130 nm range of $d_{s1}$, we observe trivial effect on the absorption properties, and the peak absorption decreases from the *perfect* condition (100% absorption) to 99% (with increasing $d_{s1}$). In the above discussed results, we chose the capping layer thickness as 100 nm, in order to balance the absorption peaks in the visible and IR regimes.

**Figure 8:**
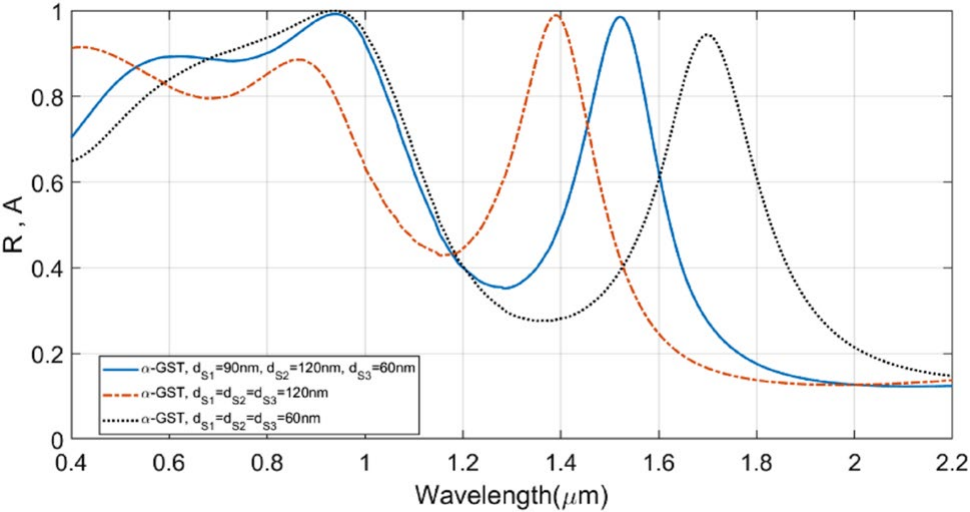
Plots of absorbance against wavelength for different values of ${\text{d}}_{{{\text{s}}1}}$, ${\text{ d}}_{{{\text{s}}2}}$, and ${\text{ d}}_{{{\text{s}}3}}$. The solid blue line corresponds to the optimum design with ${\text{d}}_{{{\text{s}}1}} = 90$ nm, ${\text{d}}_{{{\text{s}}2}} = 120$ nm, and ${\text{d}}_{{{\text{s}}3}} = 60$ nm; the dashed red line corresponds to the case of ${\text{d}}_{{{\text{s}}1}} = {\text{d}}_{{{\text{s}}2}} = {\text{d}}_{{{\text{s}}3}} = 120$ nm; the dotted black line represents the case of ${\text{d}}_{{{\text{s}}1}} = {\text{d}}_{{{\text{s}}2}} = {\text{d}}_{{{\text{s}}3}} = 60$ nm.

At this point, one would be interested to observe the effect on the absorption characteristics when all the three SiO_2_ layers in the structure are the same. That is, the parameters $d_{s1}$, $d_{s2}$ and $d_{s3}$ assume equal values. It has been comprehensively explained above that the parameter $d_{s1}$ remains useful mostly in tuning the absorption in the UV and visible regimes, whereas the values of $d_{s2}$ and $d_{s3}$ affect the absorption in the visible and mid-IR regimes. Furthermore, the parameter $d_{s3}$ is used to fine-tune the absorption peak in the mid-IR range. By making all the three parameters equal, the *perfect* absorption characteristic is lost, as becomes evident from Fig. 8. This figure illustrates the results in respect of three different designs. We notice that, in the case of $d_{s1} = d_{s2} = d_{s3} = 120$ nm, the absorption is decreased in the visible regime, while the peak in the mid-IR range is blue-shifted to 1.4 µm. On the other hand, the case of $d_{s1} = d_{s2} = d_{s3} = 60$ nm yields reduced absorption in the UV regime, and also, the same happens in the mid-IR span, where the absorbance dropped from the *perfect* absorption condition, and it is further red-shifted. Figure 8 clearly shows that the use of parametric values as $d_{s1} = 90$ nm, $d_{s2} = 120$ nm, and $d_{s3} = 60$ nm yields the best absorption performance.

The efficiency of graphene microheaters can be enhanced by reducing the contact resistance between the electrodes and graphene sheets. This ultimately reduces the total voltage required, and also, avoid the current saturation to reach high temperatures^42^. Several approaches can be exploited to reduce the contact resistance, such as patterning the electrodes and implementing the one-dimensional (1D) edge coupling^37,41^. Furthermore, the hexagonal boron nitride encapsulated graphene devices or multilayer graphene structures can be used to increase the carrier density of graphene^42^. In the present work, however, we use multilayer graphene sheets (i.e., the trilayer graphene) to increase the carrier density as well as to avoid the high voltage requirement.

**Figure 9:**
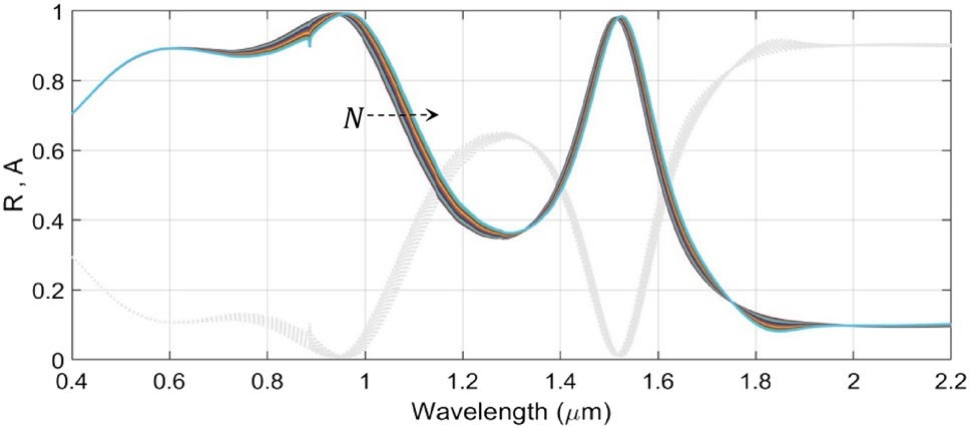
Effect on the absorption/reflection spectra due to altering the number of graphene layers in the range of 1 to 10 under fixed values of ${\text{d}}_{{{\text{GST}}1}}$ (= 130 nm), ${\text{d}}_{{{\text{GST}}2}}$ (= 70 nm), ${\text{ d}}_{{{\text{s}}1}}$ (= 100 nm), ${\text{d}}_{{{\text{s}}2}}$ (= 120 nm), and ${\text{d}}_{{{\text{s}}3}}$ (= 60 nm). The dashed arrow represents increase in the value of ${\text{N}}$.

Since we are exploiting the trilayer graphene sheets as microheaters, it would be interesting to study the effect of graphene on the optical properties of the structure. In such an attempt, we now increase the number of graphene sheets from 1 to 10 layers—the situations that correspond to the use of monolayer graphene to graphite; Fig. 9 illustrates the obtained results. We observe from this figure that gradual increase in the number of graphene layers hardly leaves significant impact on the absorption spectra in either of the two wavelength regimes (i.e., visible or IR), apart from introducing small red-shifts in the absorption peaks. This is attributed to the nearly transparent nature of graphene with very low-loss property in the stated span of wavelength.

**Figure 10:**
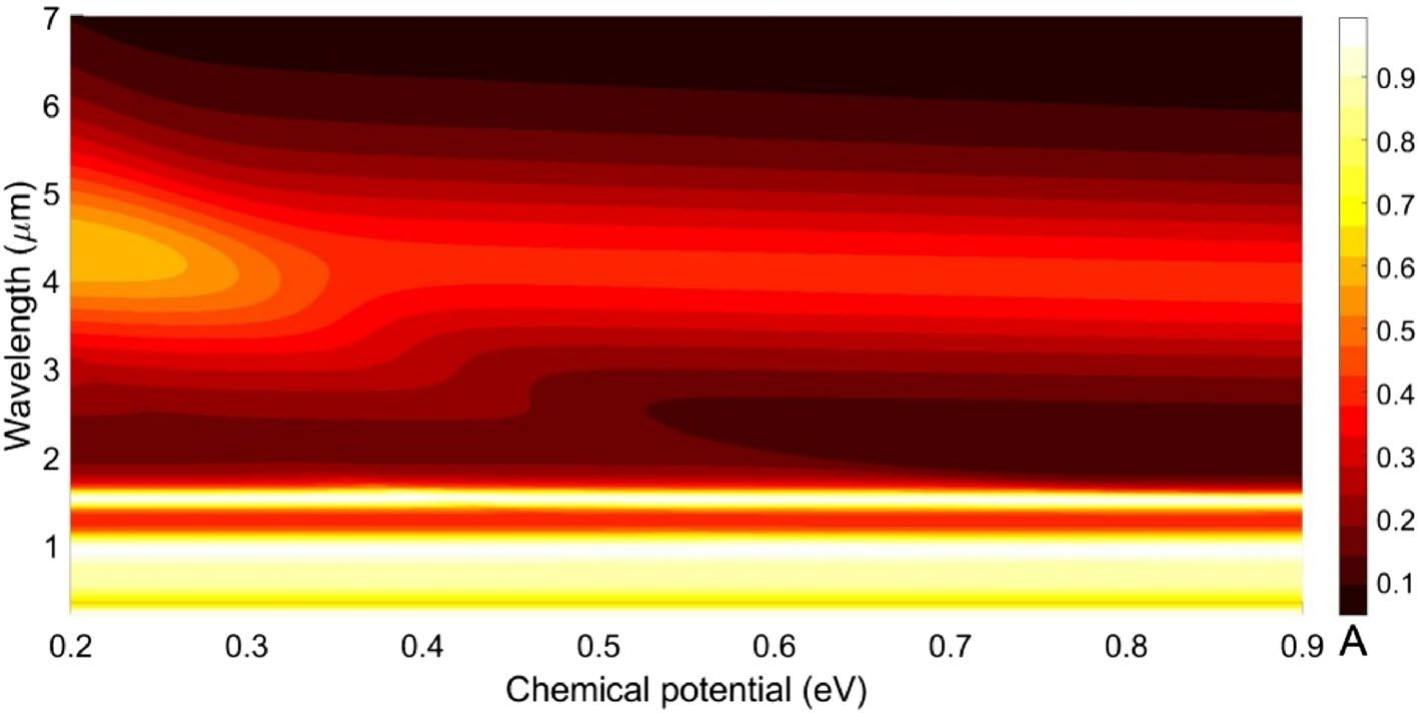
Effect on the absorption spectra due to altering chemical potential of graphene, while the other parametric and operational conditions are used as in Fig. 9.

To further study the effect of graphene layers on the absorption spectra, we now observe the impact of chemical potential $\mu_{c}$, which we vary in a range of 0.2–0.9 eV; Fig. 10 exhibits the obtained results. This figure clearly indicates that the absorption spectra in both the visible and IR regimes are not much affected due to alterations in $\mu_{c}$. However, the absorption peak at 4.4 µm wavelength (in the mid-IR regime) decreases fast from 0.7 to until it stays steady at 0.4. As such, we find that the absorption properties of the proposed structure are not enough affected by either changing the number of layers or the chemical potential. This confirms that the use of trilayer graphene mediums in the structure can serve as microheaters without imposing any disruptive effect on the optical characteristics. In other words, the proposed structure would accept applied potentials or any extra layers to generate more heat while maintaining the optical properties of the same.

**Figure 11:**
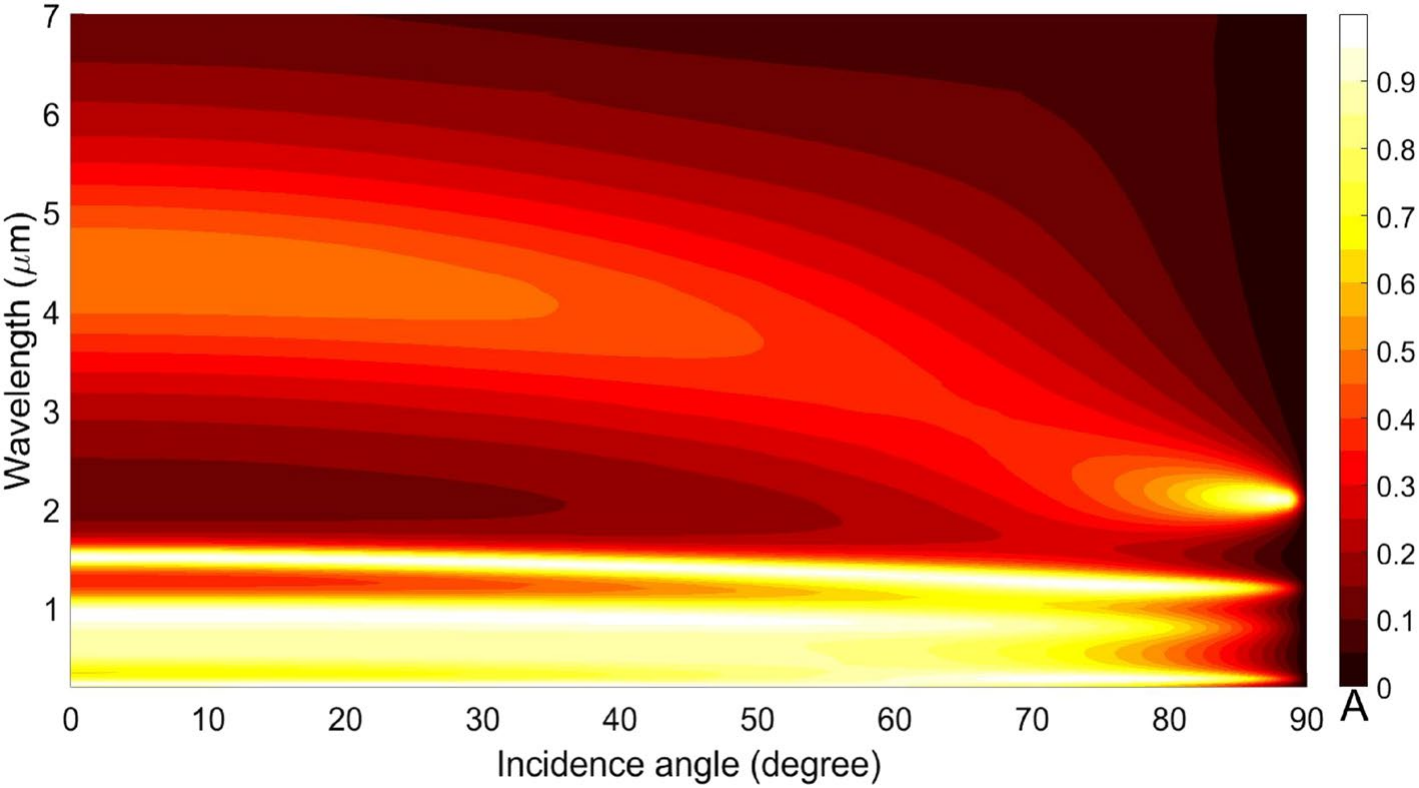
Absorption spectra of the structure under different values of incidence obliquity, while the other parametric and operational conditions are as used in Fig. 9.

So far, we discussed the results in respect of the absorption/reflection spectra of the structure under the normal incidence (i.e., $\theta = 0^\circ$) of waves impinging on the top surface (Fig. 1). We now move to the cases of oblique incidence when the obliquity varies from 0° to 90° (the grazing condition). Using $\mu_{c} = 0.1$ eV, Fig. 11 shows the obtained results; the other parametric/operational conditions are kept unchanged, as used before. We observe in this figure that, as the incidence angle increases, the absorption peaks in the visible regime are obtained corresponding to the angles < 80°, whereas the peaks in the IR regime undergo small shifts toward smaller wavelengths, as the obliquity becomes almost grazing. We also observe in Fig. 11 that the absorption peak at 4.4 µm disappears at the angles larger than 67° and another peak begins to appear from this angle onward. This clearly indicates that the absorption properties in the visible and IR regimes are not enough dependent on the incidence angle, while the absorption wavelengths can be slightly tuned.

We finally study the thermal tunability of the structure, which provides multi-level adjustments of the absorption wavelength by manipulating the refractive index (RI) of GST layer(s) in both the $\alpha$- and *c*-phases. In fact, the reversible switching between the two phases of GST mediums requires a strict control over the heating process. The permittivity of GST layer for different ratios of crystallinity can be stated by the effective permittivity theory and Lorentz-Lorenz relations^37–39^:

8 $$\frac{{\epsilon_{eff} \left( \lambda \right) - 1}}{{\epsilon_{eff} \left( \lambda \right) + 2}} = m\frac{{\epsilon_{c} \left( \lambda \right) - 1}}{{\epsilon_{c} \left( \lambda \right) + 2}} + \left( {1 - m} \right)\frac{{\epsilon_{a} \left( \lambda \right) - 1}}{{\epsilon_{a} \left( \lambda \right) + 2}}$$

9 $$n_{eff} \left( \lambda \right) + jk_{eff} \left( \lambda \right) = \sqrt {\epsilon_{eff} \left( \lambda \right)}$$

Here $m$ is the ratio of crystallinity which can take values from 0 to 1 corresponding to the pure amorphous (i.e., $m = 0$) and completely crystalline (i.e., $m = 1$) states, respectively. Also, $\epsilon_{eff} \left( \lambda \right)$, $\epsilon_{c} \left( \lambda \right)$, and $\epsilon_{a} \left( \lambda \right)$ are, respectively, the wavelength-dependent effective permittivity of GST medium, and its permittivity values in *c*- and $\alpha$-states.

**Figure 12:**
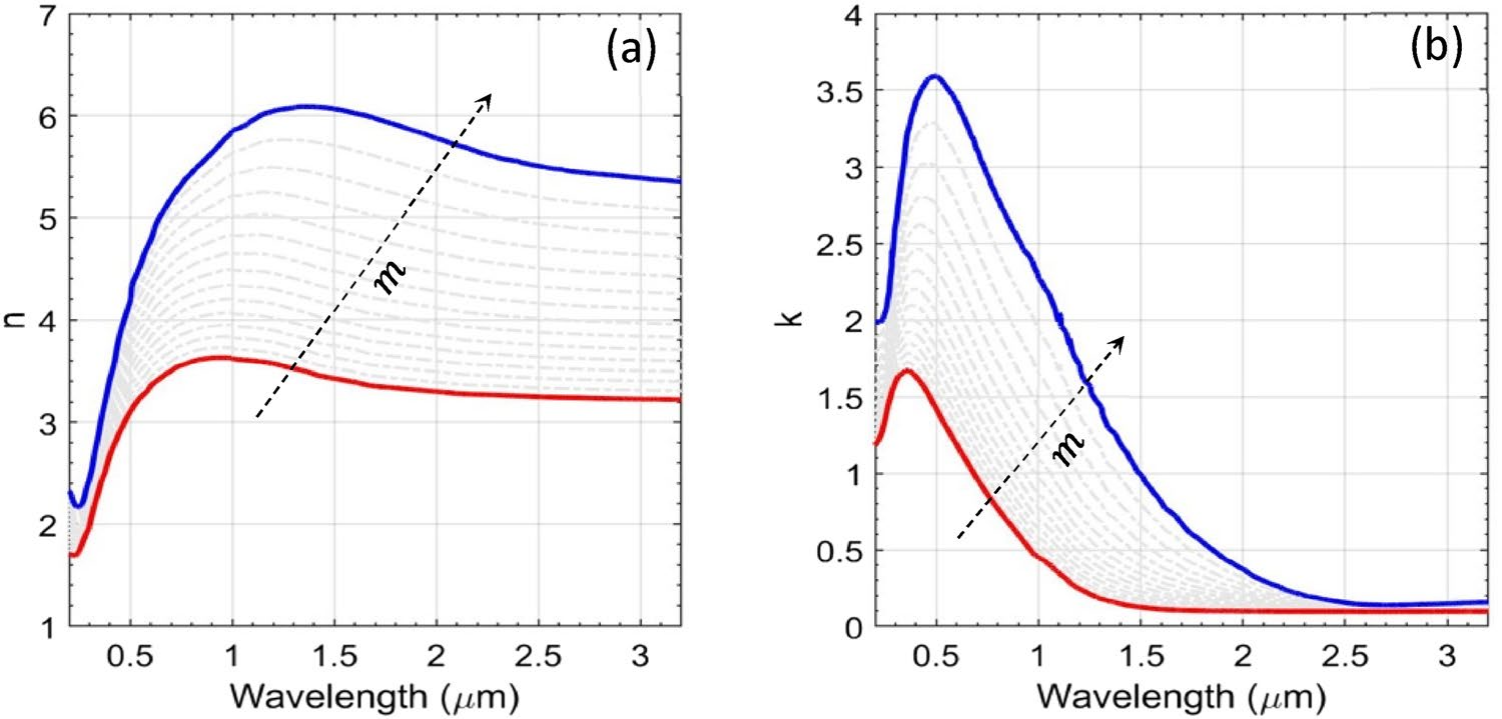
Plots of wavelength-dependent RI ${\text{n}}$ (**a**) and extinction coefficient ${\text{k}}$ (**b**) for different values of ${\text{m}}$ with $0 \le {\text{m}} \le 1$—the ${\upalpha }$-state (red solid line), the *c*-state (blue solid line), and intermediate states (gray dotted lines). The direction of dashed arrows indicates increasing ${\text{m}}$.

The wavelength dependence of the real ($n_{eff} \left( \lambda \right))$ and imaginary ($k_{eff} \left( \lambda \right))$ parts of RI of a heated GST layer can be obtained by exploiting Eq. (9); Fig. 12 exhibits the obtained results. This figure shows the RIs and extinction ratios for the amorphous ($m = 0$; solid red lines), crystalline ($m = 1$; solid blue lines), and the intermediate states (of GST) corresponding to different values of $m$ with $0 < m < 1$ (gray dashed lines). As can be seen, by increasing the value of $m$ (as indicated by the dashed arrows in Fig. 12; the direction of arrows being the indication of increasing $m$), the RI gradually moves from the $\alpha$-state (i.e., $m = 0$) toward the *c*-state (i.e., $m = 1$). This determines that, as far as the exhibited changes of GST layer is nonvolatile, the effect of Joule heating can be approximated by the level of crystalline-to-amorphous ratio.

**Figure 13:**
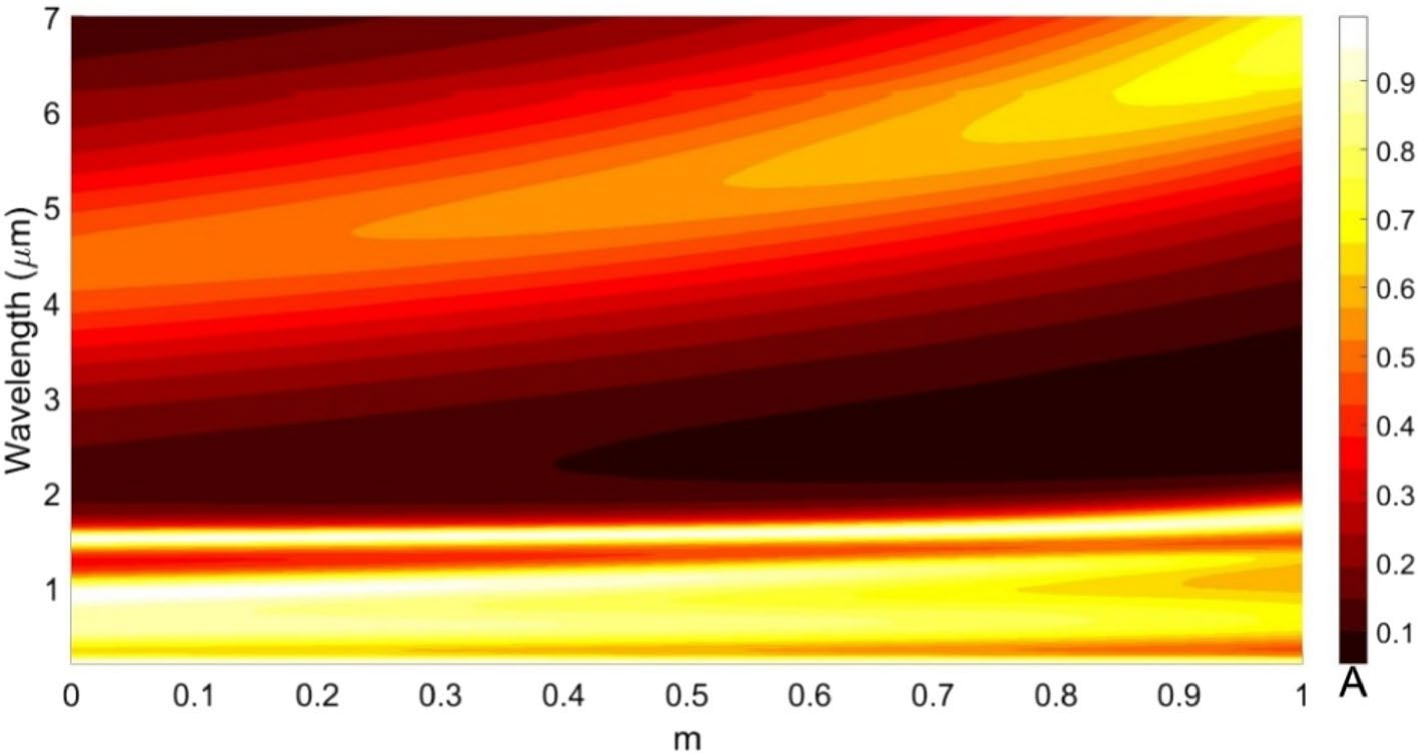
Absorption spectra for different crystalline-to-amorphous ratios of the upper GST layer, while the other parametric and operational conditions are as used in Fig. 9.

The afore discussed results correspond to the cases when the design/geometry of the proposed structure is fixed. We now attempt to introduce further tunability by independently altering the RI of GST layers in the presence of graphene microheaters. In this stream, we first consider varying the RI of the upper GST layer by sweeping the ratio $m$ from 0 to 1, and evaluate the wavelength dependence of absorption spectra in the range of visible to the mid-IR regime; Fig. 13 exhibits the obtained results when the lower GST layer remains in the $\alpha$-state.

We clearly observe in Fig. 13 that $m = 0$ results in absorption peaks in the visible and IR (~ 1520 nm) regimes. Another absorption peak with relatively less amplitude forms in the mid-IR regime around 4.67 µm. By increasing the crystallinity ratio, say for $m = 0.5$, absorption spectra shift a little toward the IR regime, whereas in the visible span, these become more expanded (as compared to the IR regime). The heating of the upper GST layer would increase $m$, which causes ultimately splitting of the absorption peaks in two parts positioned at 770 nm and 1340 nm (for $m > 0.8$; Fig. 13). The peak absorption at ~ 1520 nm, as observed before, now undergoes red-shift to 1750 nm, while maintaining the perfect absorption. In the mid-IR regime, we notice broad-band *perfect* absorption at ~ 6500 nm. As such, we see that the heating of the upper GST layer results in shifting the absorption peaks from the IR to the mid-IR regime without significantly affecting the magnitude of absorption. However, such an operation causes expansion of absorption spectra in the visible regime, as long as $m < 0.7$.

**Figure 14:**
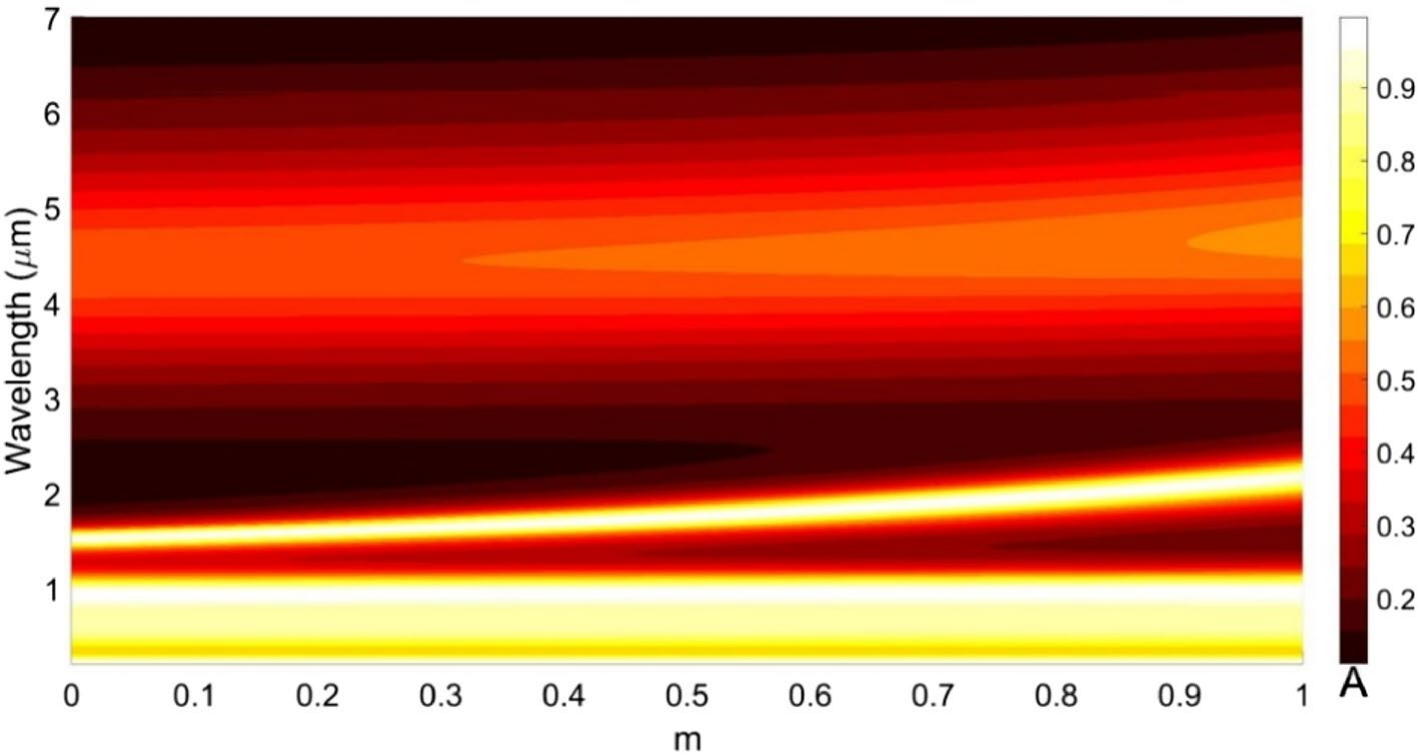
Absorption spectra for different crystalline-to-amorphous ratios of the lower GST layer, while the other parametric and operational conditions are as used in Fig. 9.

Figure 14 illustrates the effect of heating (i.e., varying $m$ from 0 to 1) the lower GST layer, considering the upper GST layer in the $\alpha$-state. We notice that the absorption spectra in the visible wavelength span remain unchanged including the peak absorption values, whereas those in the IR span experience red-shifts in resonance wavelengths from 1520 to 2184 nm along with slight broadening in the absorption bandwidth. This determines that the lower GST layer remains more responsible to alter the spectral characteristics in the IR regime. However, the presence of weak absorption peak in the mid-IR region is attributed to the RI of the upper GST layer in the structure. As such, the investigation reveals that the absorption spectra in the visible regime are better controlled by the upper GST layer, whereas those in the IR (or the mid-IR) span are governed more by the lower GST layer.

Table 1 presents the comparative aspects (in terms of performance characteristics and the other operational and/or geometrical features) of the proposed GST-graphene-based absorber structure with some of the previously reported works^52–56^. We clearly observe that the proposed structure offers a broad absorption bandwidth with simultaneously maintaining the stability (in absorption characteristics) against the incidence obliquity of waves. A wide range of absorption in either the visible or mid-IR regime can be governed by controlling the crystallinity ratio of GST layers, while the thickness of the structure is comparable with other multilayer kind of absorbers.

**Table 1:** Comparison of the features of the previously reported metamaterial absorbers with the proposed GST-graphene-based structure.

Absorber structure	Tuning range	Angular stability	Periodicity	Thickness (nm)	Experimental verification
Patterned metasurface-based^52^	300–450 nm	0–45°	133 nm	399	Yes
Multilayered stack-based^53^	450–750 nm	0–50°	N/A	417	Yes
Patterned metasurface-based^54^	400–650 nm, 1200–1600 nm (tunable)	0–60°	600 nm	800	No
Patterned metasurface-based^55^	400–750 nm	0–60°	200 nm	65	No
Multilayered stack-based^56^	350–900 nm	0–75°	N/A	350	Yes
Multilayered stack-based (the present work)	350–1340 nm, 1520–2184 nm (tunable)	0–75°	N/A	532	No

## Conclusion

The investigation is pivoted to the optical response of specially designed multilayered structure comprised of dielectric, GST alloys in amorphous and crystalline states and trilayer graphene sheets. The results reveal the presence of absorption spectra in the visible and IR regimes. Under different parametric and operational conditions, the incorporation of two GST layers in the structure results in perfect absorption. The width and position of absorption bands can be governed by suitably controlling the layer thicknesses of different mediums and incidence obliquity. The embedded graphene microheaters can be utilized to achieve phase transition of GST layers, thereby introducing additional tunability feature of the structure. It has been found that the graphene layer has no disruptive effect on the optical properties as the relevant functional parameters, namely the number of sheets (in the structure) and chemical potential, do not affect the spectral performance. The results demonstrate that the use of 10 graphene layers and 0.9 eV chemical potential slightly improves absorption. The GST crystallization process has been simulated by introducing the crystalline-to-amorphous ratio—the feature that adds more tunability of the absorption spectra. The obtained results indicate the usefulness of the structure in various photonic applications, such as bolometry, label-free biosensors, IR detectors, and tunable wide-band absorbers.
